# Neurogenic and Myogenic Properties of Pan-Colonic Motor Patterns and Their Spatiotemporal Organization in Rats

**DOI:** 10.1371/journal.pone.0060474

**Published:** 2013-04-05

**Authors:** Ji-Hong Chen, Qian Zhang, Yuanjie Yu, Kongling Li, Hong Liao, Longying Jiang, Lu Hong, Xiaohui Du, Xinghai Hu, Sifeng Chen, Sheng Yin, Qingmin Gao, Xiangdong Yin, Hesheng Luo, Jan D. Huizinga

**Affiliations:** 1 Department of Gastroenterology and Hepatology, Renmin Hospital of Wuhan University and Wuhan University Institute of Digestive and Liver Diseases, Wuhan, Hubei, China; 2 Department of Electronics and Information Engineering, Huazhong University of Science and Technology, Wuhan, Hubei, China; 3 Farncombe Family Digestive Health Research Institute, Department of Medicine, McMaster University, Hamilton, Canada; Temple University School of Medicine, United States of America

## Abstract

**Background and Aims:**

Better understanding of intrinsic control mechanisms of colonic motility will lead to better treatment options for colonic dysmotility. The aim was to investigate neurogenic and myogenic control mechanisms underlying pan-colonic motor patterns.

**Methods:**

Analysis of *in vitro* video recordings of whole rat colon motility was used to explore motor patterns and their spatiotemporal organizations and to identify mechanisms of neurogenic and myogenic control using pharmacological tools.

**Results:**

Study of the pan-colonic spatiotemporal organization of motor patterns revealed: fluid-induced or spontaneous rhythmic propulsive long distance contractions (LDCs, 0.4–1.5/min, involving the whole colon), rhythmic propulsive motor complexes (RPMCs) (0.8–2.5/min, dominant in distal colon), ripples (10–14/min, dominant in proximal colon), segmentation and retrograde contractions (0.1–0.8/min, prominent in distal and mid colon). Spontaneous rhythmic LDCs were the dominant pattern, blocked by tetrodotoxin, lidocaine or blockers of cholinergic, nitrergic or serotonergic pathways. Change from propulsion to segmentation and distal retrograde contractions was most prominent after blocking 5-HT_3_ receptors. In the presence of all neural blockers, bethanechol consistently evoked rhythmic LDC-like propulsive contractions in the same frequency range as the LDCs, indicating the existence of myogenic mechanisms of initiation and propulsion.

**Conclusions:**

Neurogenic and myogenic control systems orchestrate distinct and variable motor patterns at different regions of the pan-colon. Cholinergic, nitrergic and serotonergic pathways are essential for rhythmic LDCs to develop. Rhythmic motor patterns in presence of neural blockade indicate the involvement of myogenic control systems and suggest a role for the networks of interstitial cells of Cajal as pacemakers.

## Introduction

Transit, absorption of nutrients, salts, vitamins and water; storage, stool shaping and excretion are major functions of the colon that may involve specialized colonic motor functions, which are not fully understood. A more comprehensive insight into colonic motor patterns including propulsive and non-propulsive activities and their control mechanisms is needed to identify possible biomarkers of colonic motility disorders. Although many *in vivo* and *in vitro* studies have shed light on mechanisms of colonic motor activity, high-resolution techniques are markedly increasing our ability to study essential details of motor patterns. Using such techniques, cooperation between neurally-induced pacemaker activity by interstitial cells of Cajal (ICC) and enteric neural programs were hypothesized to control colonic propulsive motor patterns in rats [1] and mice [2], suggesting that division of motor activities or motor dysfunction into exclusively neurogenic or exclusively myogenic may not reflect the reality of gut motility control. The present study pursues further insight into the integration of neurogenic and myogenic control mechanisms of propulsive and non-propulsive motor activities. Patients with slow transit constipation showed a markedly abnormal colonic motor pattern with paucity of propagating pressure waves in the mid colon and increase in frequency of retrograde propagating sequences in the proximal colon, suggesting the importance of antegrade long distance contractions in normal colonic transit [3], which is the focus of the present study. Different regions of the colon have different functions and therefore may show different dominant motor patterns under physiological and pathophysiological conditions. These patterns, their control mechanisms and in particular the interaction of these patterns can only be observed in pan-colonic studies hence we investigated the spatiotemporal organization in the whole colon *in vitro*. The focus of the present study was the investigation of rhythmic pan-colonic propulsive motor patterns, before and after nerve blockade to explore the potential roles of myogenic control systems including ICC and the enteric nervous system.

## Materials and Methods

The whole colon was examined from 55 adult male Sprague-Dawley rats weighing 150–300 g. Animals were killed by cervical dislocation. The entire colon was removed and placed in gassed (3% CO_2_ and 95% O_2_ (v/v)) Krebs solution (pH 7.3–7.4) at 37°C. Krebs solution consisted of (mM) NaCl 118.1, KCl 4.8, NaHCO_3_ 25, NaH_2_PO_4_ 1.3, MgCl_2_ 6 H_2_O 1.2, glucose 12.2 and CaCl_2_ 2.5. The contents of the colon were gently flushed out using warmed Krebs solution and the external connective tissue was removed. The distal and proximal ends were cannulated and fixed to the bottom of the organ bath. The inflow tube at the proximal end was connected to a reservoir (a 50 ml syringe) placed 15 cm above the level of the colon with PBS (phosphate buffered solution with 10 µM indomethacin, without glucose). The inflow tube (inner diameter 3 mm, outer diameter 4 mm) was filled with PBS, no air, and a valve was situated 5 cm above the colon that was normally closed but was opened when fluid was allowed to enter the colon by gravity. The outflow tube (inner diameter 3 mm, outer diameter 4 mm) was positioned in a narrow upright container filled with PBS. The fluid level in the container determined the intraluminal pressure and could be adjusted by raising or lowering the fluid level. The standard intraluminal pressure at the beginning of the experiment was 5 cm H_2_O. Propulsive contractions were identified by the rising fluid level in the narrow container. After an LDC was completed, fluid flowed back into the colon. The colon was left to equilibrate for 20–30 minutes before the experiment started. A video camera was mounted above the preparation and each experiment was recorded in its entirety. Data acquisition occurred through a Microsoft camera using Microsoft Lifecam software. Video recordings were analyzed using Image J aided by plug-ins written by Dr Sean Parsons [1]. A “spatiotemporal map” is an image representation of motor activity based on the changes of the colon width. Diagonal streaks of dark color represent propagating contractions. Colon width (coded as image intensity, black to white) was calculated at each point (pixel) along the colon's length and changes over time displayed. Changes in diameter were quantified after calibrating distance, using dots at the bottom of the organ bath, which were separated by exactly 1 cm. The diameter of the colon was 0.83±0.17 cm (n = 55). Units of diameter change in table are in cm. Statistics were carried out using the Student’s t test for paired or unpaired data. The duration of a propulsive contraction (LDCs and other types) was determined by identifying the beginning of the contraction and the end of the relaxation phase using both the spatiotemporal map and the video. If there was a slight difference between the two, we took the average of the two values. Data were expressed as mean±SD.

The maps were made of the whole colon except for the most proximal and most distal parts that were used to fix the colon onto in and outflow tubes. The sections not visible in the maps were between 1.3 and 1.9 cm in length.

This study was carried out in strict accordance with the recommendations in the Guide for the Care and Use of Laboratory Animals of the National Institutes of Health. The protocol was approved by the ethics committee of Renmin Hospital of Wuhan University (NSFC-81170249).

Physiological salt solution (PBS) (phosphate buffer without glucose) was used for fluid infusion to stimulate the colon. The fluid reservoir was situated 15 cm above the colon and the fluid stream (2–3 ml) lasted 8.8±3.2 seconds. PBS consisted of (mM) NaCl 137.1, KCl 2.7, Na_2_HPO_4_ 10, KH_2_PO_4_ 2.

The following drugs were used at the final bath concentrations indicated: 100 µM lidocaine (Hualu Pharmaceutical Co. Ltd., Shangdong, China) and 0.2 µM tetrodotoxin (TTX; Baoman Biochemistry Co. Ltd., Shanghai, China) were used to block neural activity. 200 µM nitro-L-arginine (L-NNA; from Aladdin Chemistry Co. Ltd., Shanghai, China) was used to inhibit nitric oxide synthesis. 5-HT_3_ antagonist granisetron (NingBo Team Pharm Co., Ltd., China) was used to block 5-HT_3_ receptors. 2 µM atropine (from Aladdin Chemistry Co. Ltd., Shanghai, China) was used to block muscarinic acetylcholine receptors. 2 µM bethanechol (3B Scientific Corporation, Libertyville, Illinois, USA) or carbachol 2 µM (Shandong Chia Tai Freda Pharmaceutical Group) was used to evoke activity after nerve blockade. Krebs reagents were purchased from Sinopharm Chemical Reagent Co., Ltd., Shanghai, China.

## Results

We observed distinct colonic motor patterns in the fluid filled whole rat colon: long distance contractions (LDCs), interrupted LDCs, tandem contractions, rhythmic propulsive motor complexes (RPMCs), propagating ripples, segmentation and retrograde contractions. Their generation was not solely myogenic or neurogenic. Several of these motor patterns occurred predominantly in one region of the colon.

### Long Distance Contractions (LDCs)

**Figure 1:**
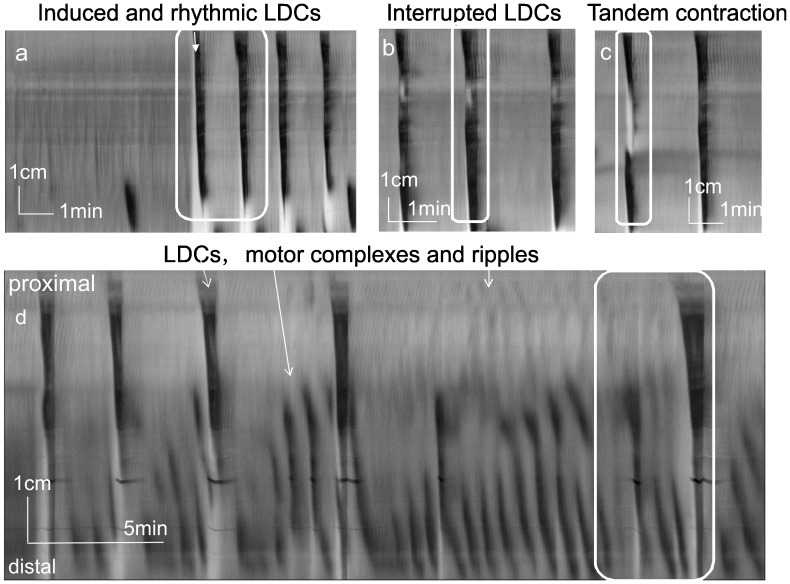
Propulsive motor patterns. Spatiotemporal maps created from video recordings of motor patterns of the whole rat colon. The colon as well as the in and outflow tubes are filled with PBS and the fluid column in the outflow tube determines an intraluminal pressure of 5 cm H_2_O in the experiments described in this figure and all other figures. a. In a quiescent colon, fluid-infusion (at white arrow, see methods) induced a long distance contraction (LDC) which was followed without further stimulus by rhythmic LDCs. Ripples followed the LDCs in the proximal colon. Boxed area = Video S1 “Induced and spontaneous LDC”. The white band preceding the first LDC is the distention caused by fluid infusion. The white areas preceding spontaneous LDCs are relaxations preceding the contraction. b. Interrupted LDCs showed a relaxation at about 1/3 down the colon length. The interruption (relaxation) was seen as a white spot on the black contraction. The boxed LDC is shown in Video S2 “Interrupted LDC”. c. A tandem contraction (see text) is shown followed by a spontaneous LDC. Boxed area = Video S3 “Tandem contraction” d. Five rhythmic LDCs are shown together with RPMCs. Ripples were seen in the proximal colon. The boxed area is shown in Video S4 “Motor complexes and LDC”.

The LDC had 2 components, a contraction phase lasting ∼ 30 s which was followed by a relaxation phase lasting ∼10 s (Figure 1, Table 1, Video S1). The LDC started in the most proximal colon and propagated most often to the distal end but covered at least 2/3 of the colon (Table 1); the forceful circumferential contraction moved all the intraluminal content in anal direction, out of the colon into the outflow reservoir. The contraction was preceded by a relaxation (white band in spatiotemporal map) starting 1/4–1/3 down the colon. The LDC was not a ring contraction since the colon remained contracted following the propagating front of the contraction. The contraction was followed by the relaxation phase that was characterized by a complete and sudden relaxation of the whole contracted colon; the wave of relaxation started in the most distal part of the colon and propagated in oral direction. The intraluminal liquid content would flow back into the whole colon in our standard open outflow experimental setup. The content would also flow back when the outlet was obstructed. In spatiotemporal maps, the contraction phase of the LDC showed as a black elongated triangle (Figure 1). LDCs occurred spontaneously in a rhythmic manner at 0.64±0.17/min. In a quiescent colon, rhythmic LDCs could invariably be induced by fluid infusion into the proximal colon, occurring within 1–5 s of the start of infusion. Fluid infusion-induced LDCs had similar characteristics as the spontaneous LDCs (Figure 1a, Table 1, Video S1).

**Table 1:** Characteristics of the propulsive colonic motor patterns.

	Long distance contractions (LDCs)			Tandem contraction		In presence of nerve blockade (TTX or lidocaine) plus bethanechol	
	Spontaneous (n = 26)	Induced (n = 13)	Interrupted (n = 11)	First part (n = 14)	Second part (n = 14)	LDC-like motor pattern (n = 6)	Retrograde contractions (n = 6)
Propagation length (cm)	10.0±2.0	11.5±1.9	10.6±1.2	4.3±1.5	5.6±2.0	11.1±2.8	4.8±1.3
Contraction duration (s)	32.8±13.2	21.6±7.4	27.7±10.7	15.6±6.3	19.7±14.8	66.1±37.4**	65.6±32.7
Relaxation phase duration (s)	10.3±3.0	8.5±2.8	10.2±2.6	6.9±3.9	8.9±2.8	11.8±4.2	18.2±10.1
Frequency (cpm)	0.65±0.13	NA	0.73±0.31	0.50±0.21		0.36±0.11*	0.25±0.12
Velocity (mm/s)	3.5±1.5	5.3±2.6	4.3±1.5	3.2±1.9	3.5±1.3	2.2±1.2*	0.9±0.4
Relaxation phase velocity (mm/s)	10.4±3.7	13.5±6.8	10.9±2.9	7.7±4.3	6.6±2.4	10.1±0.4	3.4±2.2
Diameter change (cm)	0.41±0.12	0.42±0.12	0.44±0.11	0.34±0.10	0.43±0.10	0.32±0.10	0.34±0.08

Mean values ± S.D. The length of the colon was 13.8±1.8 cm; the colon diameter was 0.83±0.17 cm (n = 55). * = p<0.05, ** = p<0.0001 comparing LDC-like activity after TTX or lidocaine with spontaneous LDCs before nerve blockade. NA =  not applicable. n = number of animals.

An “interrupted LDC” developed when the relaxation that started to precede the contraction, transiently abolished the contraction in the middle of the colon with a relaxation that spanned 1.9±1.1 cm and lasted for ∼ 10 s (Table 1) whereupon the contraction started again and proceeded to the distal colon (Figure 1b, Table 1, Video S2).

The “tandem contraction” was a distinct motor pattern seen in all colons (Figure 1c, Table 1, Video S3). The pattern either started with a slowly developing propulsive contraction in the proximal colon whereupon a second contraction started to develop after 8.9±6.4 s in the mid or distal colon, or the pattern started with the distal contraction followed by the proximal contraction (interval 12.7±7.0 s). Both proximal and distal contractions propagated simultaneously in anal direction. Invariably, a relaxation developed anal to the proximal contraction and this contraction ended in the middle of the colon where the second contraction was ongoing. The content of the proximal colon entered the relaxation part of the colon and then flowed back since the distal contraction prevented it from moving to the distal colon.

### RPMCs and LDCs

Starting from the mid or distal colon, RPMCs occurred with variable frequencies of 0.5 to 2/min (average 1.6±0.4/min, n = 8) and variable velocities (4.3 to 9.2 cm/min, average 6.8±1.5 cm/min, n = 8) (Figure 1d, video S4). They propelled the content into anal direction with amplitudes as high as that of spontaneous LDCs (average lumen diameter reduction 32±8% vs 41±12%, p<0.218). The RPMCs most often did not have a relaxation preceding the propagating contraction and there was no relaxation phase upon termination of the contraction. RPMCs were seen to be propagating ring contractions without the sustained component of the LDC. Three to 8 RPMCs often occurred in between 2 LDCs (Figure 1 d) in the distal colon but could also occur in the absence of LDCs; they were often abolished by TTX, identifying them as neurogenic.

### Propagating Ripples and LDCs

Ripples are superficial ring contractions of the circular muscle that occur at a distinct frequency, similar to the frequency of the dominant slow wave and quite constant within a preparation. They have been described in the guinea pig colon [4], [5] and in the rat colon [1].

**Figure 2:**
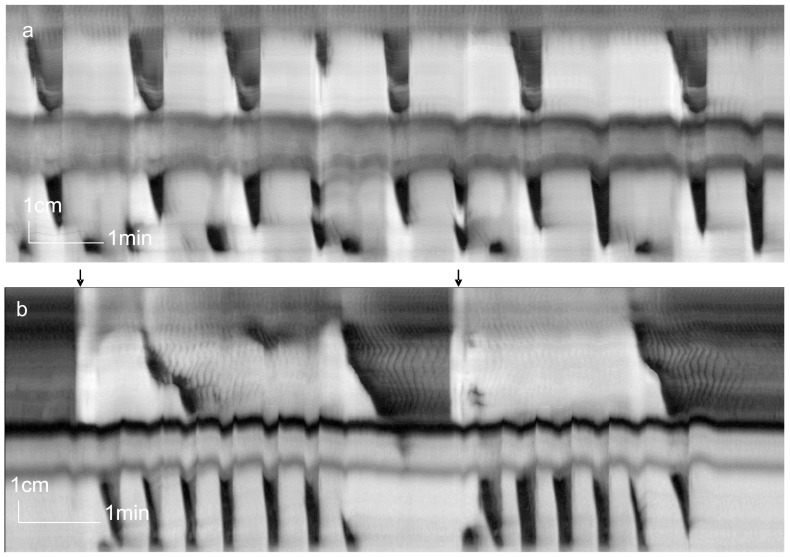
Influence of a sustained contraction/distention on the LDC. Spatiotemporal maps created from video recordings of motor patterns of the whole rat colon. a. A sustained contraction was present in the mid colon. LDCs started in the proximal colon and were interrupted by the sustained contraction but proceeded normally at the distal end of the sustained contraction. In addition, three contractions were seen that started at the distal end of the sustained contraction. They fall under the definition of RPMCs. b. A sustained distention, preceded and followed by a narrow ring contraction, was present in the mid colon. The LDCs in this experiment were exceptionally long in duration. The LDCs did not proceed after the contraction. In between the LDCs, RPMCs were initiated at the distal side of the sustained contraction.

In the present study, propagating ripples were observed as rhythmic, high frequency (ranging from 6 −12/min; 9.5±0.5/min, n = 30) superficial contractions. Diameter changes were of low amplitude at 0.07±0.02 cm (n = 30) in most parts of the colon during the whole recording time. They originated most often in the proximal colon, propagating antegrade, retrograde or in both directions (Figure 1 d). LDCs were always followed by a period of high excitation in the proximal colon, as reflected by ripples at relatively high amplitude, propagating most often retrograde but sometimes antegrade for a period of 30∼60 seconds (Figures 1E, 2A). The diameter changes were 0.08±0.01 cm after LDCs vs. 0.03±0.03 cm preceding LDCs (p<0.001).

### Sustained Narrow Bands of Constriction and The Initiation of Rhythmic Propulsive Contractions

A sustained distention, preceded and followed by narrow bands of sustained contractions, occurred spontaneously (n = 2) for the duration of the experiment (Figure 2a,b). An LDC either stopped at the preceding contraction (Figure 2b) or continued at the distal end (Figure 2a). The distension/contraction also initiated rhythmic propulsive contractions (Figure 2a,b).

### Effects of Inhibition of Neural Pathways

**Figure 3:**
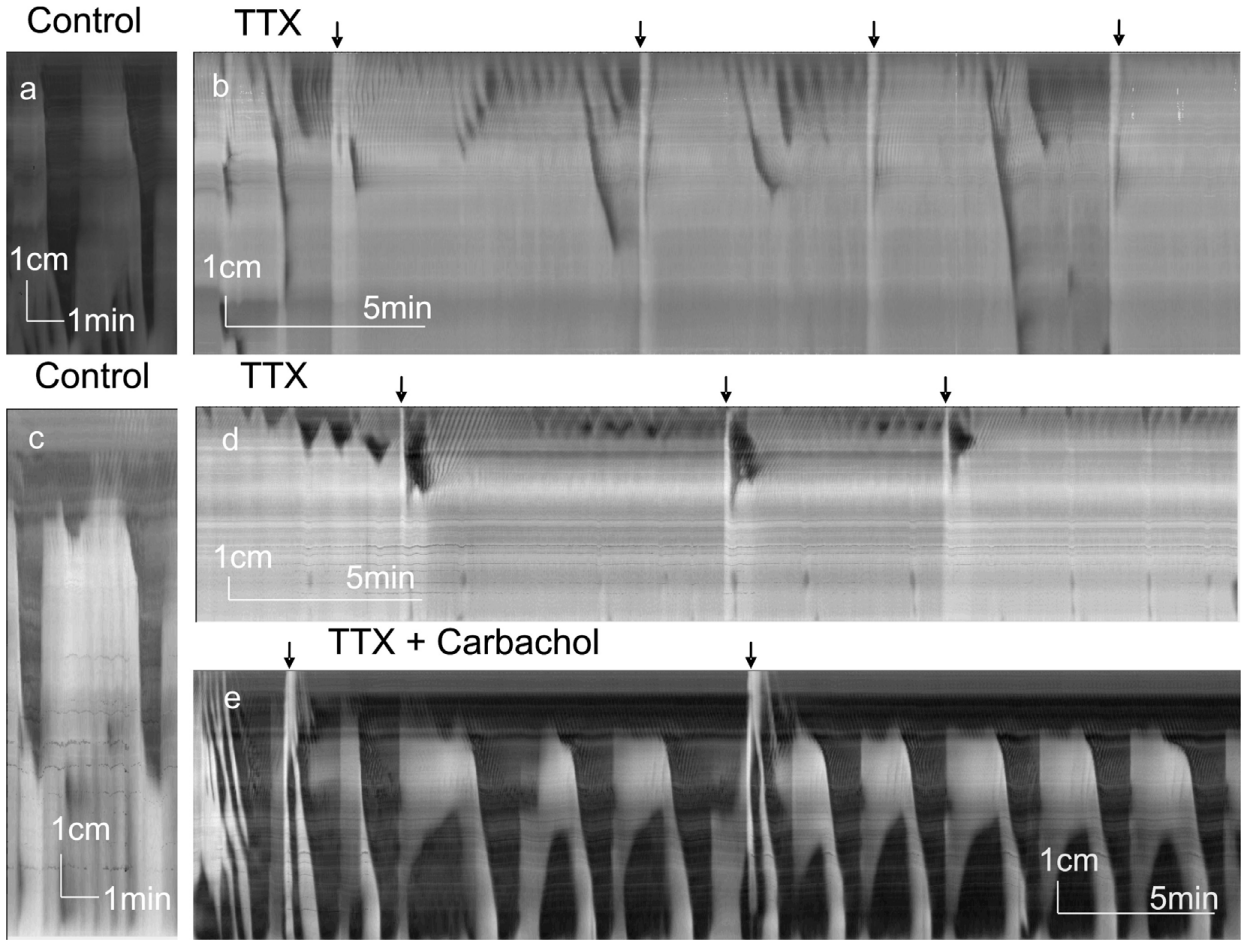
Effect of TTX on LDCs. Spatiotemporal maps created from video recordings of motor patterns of the whole rat colon. All arrows indicate infusion of PBS into proximal colon. a. Control LDCs followed by addition of TTX, added 1 min before the start of panel b. b. TTX abolished induced and rhythmic LDCs. Fluid infusion occurred at arrows. Rhythmic contractions remained in the proximal colon and a few propagated into the mid colon. c. Control LDCs (different animal). d. TTX was added 7 min before the start of panel d. TTX abolished induced and rhythmic LDCs. Rhythmic motor activity remained in the proximal colon. e. Carbachol, 2×10^−6^ M, was added 10 min before start of panel e. Carbachol, in the presence of TTX induced rhythmic LDC-like activity as well as retrograde contractions in the distal colon.

**Figure 4:**
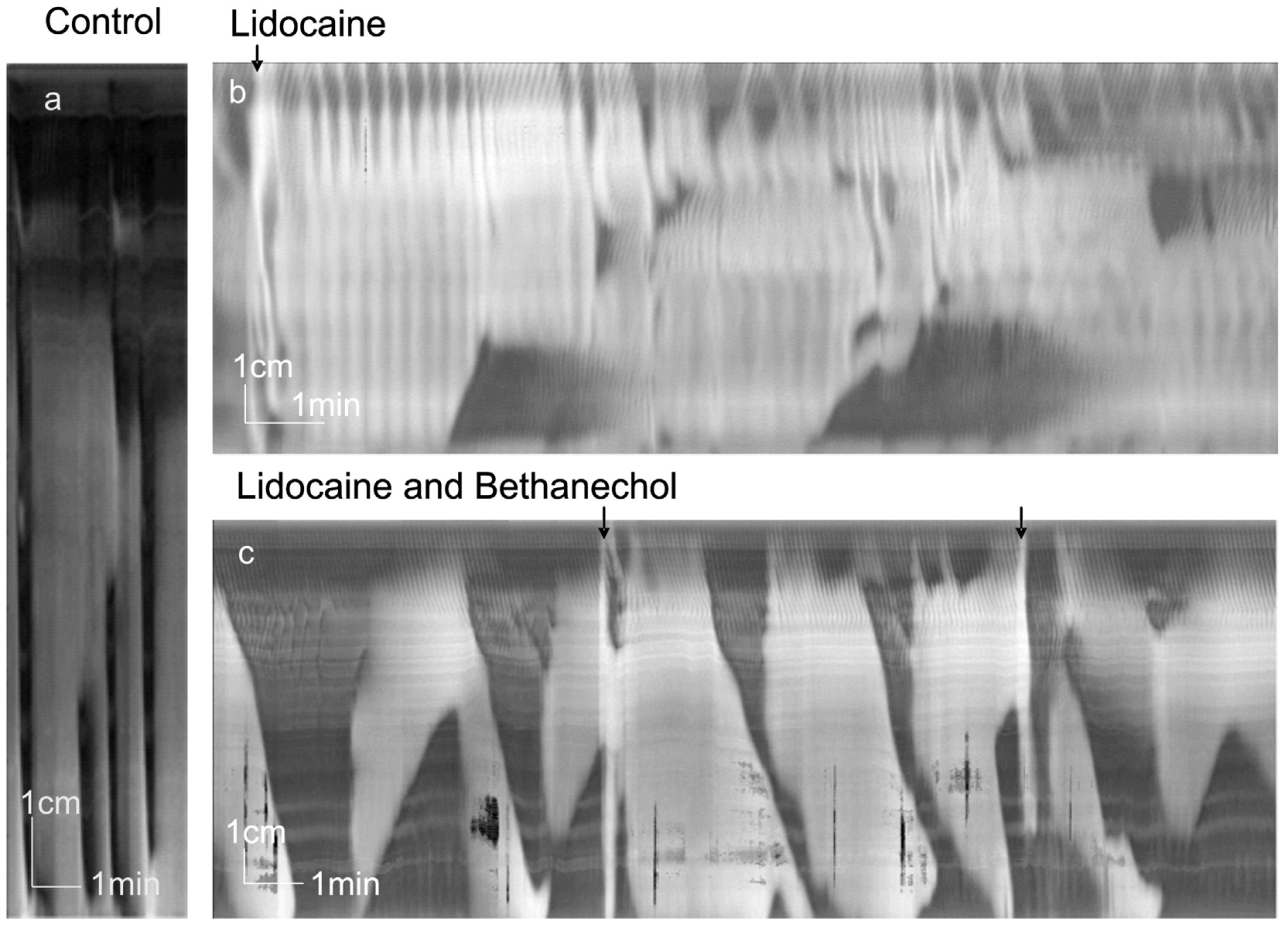
Lidocaine and bethanechol. Spatiotemporal maps created from video recordings of motor patterns of the whole rat colon. All arrows indicate infusion of PBS into proximal colon. a. Control LDCs with distal RPMCs. b. Lidocaine abolished LDCs but rhythmic contractile activity remained up to 6/min. Two retrograde contractions were seen. Lidocaine was added 3 min before start of panel b. c. Addition of bethanechol (2×10^−6^ M) introduced LDC-like activity as well as retrograde contractions in the distal colon. Fluid infusion effects were variable, sometimes without effect (first arrow), sometimes inducing a propulsive contraction. Bethanechol was added 14 min before start of panel c.

TTX (1 µM) and lidocaine (0.1 mM) completely inhibited LDCs. Fluid infusion-induced LDCs did not develop in the presence of TTX and spontaneous LDCs reduced in frequency until they were abolished after 5–30 min (n = 10, Figures 3b,d). In the presence of TTX, rhythmic contractile activity in the proximal colon most often remained, appearing similar to the starting part of the LDCs with shorter length (3.1±0.9 cm), lower change in diameter (0.12±0.05 cm) and shorter duration (∼ 22 s) (Table 1). The frequency varied from 0.6 to 2/min (average 1.1±0.6/min). The effect of lidocaine (n = 6) was similar to that of TTX with rhythmic proximal contractions remaining at variable frequencies up to 6/min (Figure 4b).

In the presence of TTX or lidocaine, carbachol (2 µM) or bethanechol (2 µM) induced strong rhythmic contractile activity (n = 8, Figures 3e, 4c, Table 1). This activity was similar to LDCs and is here described as LDC-like. The amplitude was higher, the relaxation phase was short lasting, the propagation velocity in the mid and distal colon was slower, the duration was longer and there was no increased ripple activity after the contraction (Table 1). The LDC-like activity was almost always associated with retrograde contractions in the distal colon (Figures 3e, 4c, Table 1).

**Figure 5:**
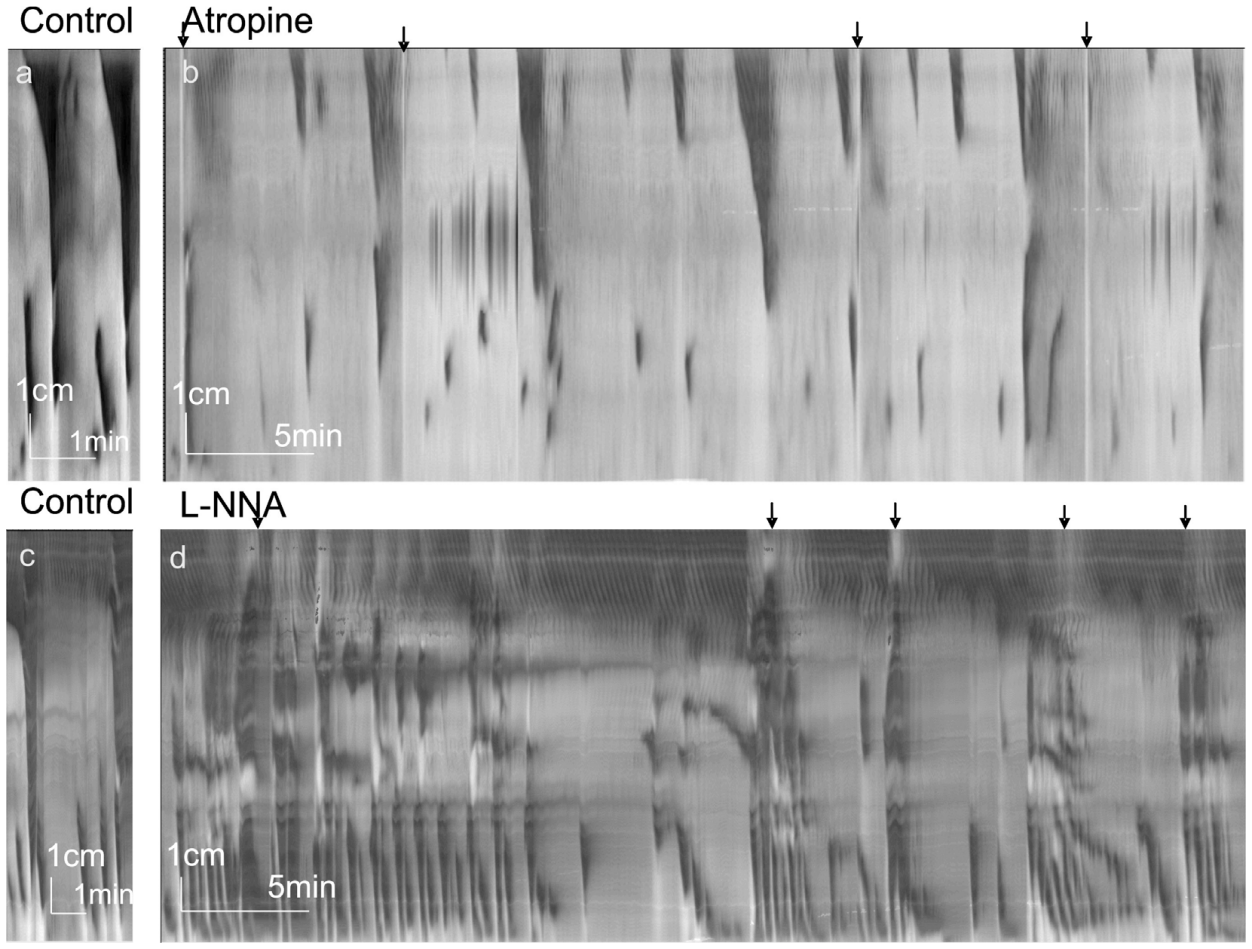
Atropine and L-NNA. Spatiotemporal maps created from video recordings of motor patterns of the whole rat colon. All arrows indicate infusion of PBS into proximal colon. a. Control LDCs with distal RPMCs. b. After addition of atropine the LDCs were getting smaller and disappeared after 30 min. Atropine was added 3 min before start of panel b. c. Control LDCs with distal RPMCs. d. Typical LDCs were not seen after blockade of nitric oxide synthesis. Strong rhythmic contractile activity was always present showing various and varying patterns at frequencies that were higher compared to control activity. L-NNA was added 8 min before start of panel d.

Atropine (2 µM) abolished LDCs evoked by fluid-induced distention (Figure 5b, n = 6). Spontaneous LDCs were inhibited: 30 min after addition of atropine their frequency was decreased from 0.48±0.11 to 0.18±0.11/min (p<0.01). After 30 min, in all experiments, rhythmic activity remained in the proximal colon and appeared to be the start of LDCs but did not propagate after 4.2±0.6 cm. The proximal contractions in the presence of atropine occurred at 0.32±0.10/min.

L-NNA (0.2 mM) abolished the LDCs (n = 5, Figure 5d). After abolishment of the LDCs, the proximal colon activity was dominated by strong ripple activity at 11.2±0.4/min, propagating over a length of 7.2±1.4 cm. The direction of ripple propagation changed frequently. The distal colon activity was dominated by RPMCs starting in the mid colon, occurring rhythmically at high frequency of 6.4±0.9/min and velocity of 8.8±3.5 cm/min. The high frequency activity in the distal colon was not observed when both atropine and L-NNA were present (n = 6). Ripples remained prominent in the presence of both atropine and L-NNA at 12.0±1.4/min, propagating over a length of 6.0±3.5 cm. The direction of propagation changed frequently.

**Figure 6:**
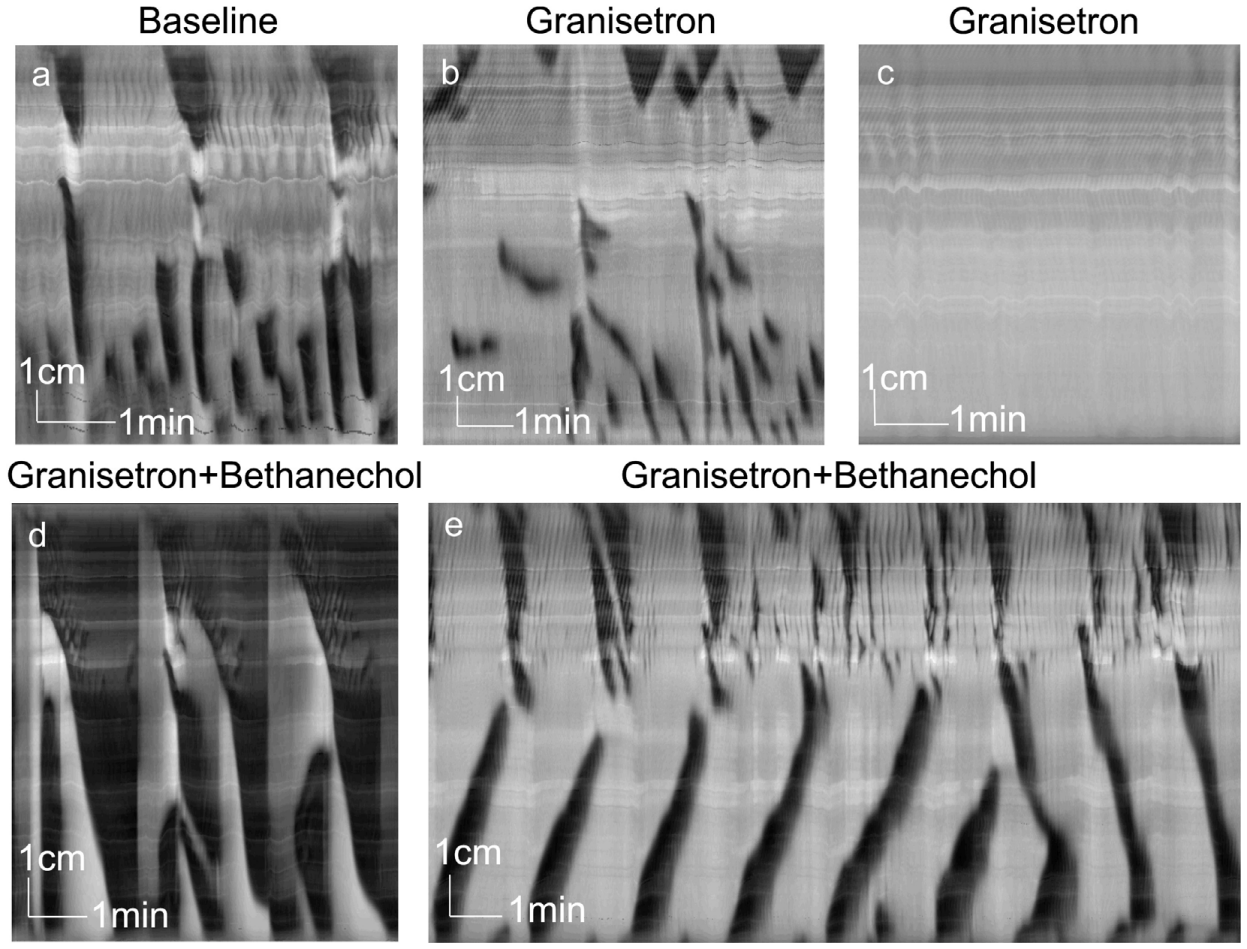
Effect of the 5-HT_3_ antagonist granisetron. Spatiotemporal maps created from video recordings of motor patterns of the whole rat colon. a. Control activity consisted of a tandem contraction and two interrupted LDCs accompanied by distal antegrade RPMCs (rat 1). b. After addition of granisetron (3.8×10^−6 ^M, added 30 min before start of panel b), LDC activity was abolished, RPMCs were disrupted and segmentation activity was induced in the mid and distal colon (rat 1). c. LDCs and related motor patterns were abolished 20 min after addition of granisetron (3.8×10^−6 ^M, rat 2) for a period of 5 min, thereafter segmentation activity emerged (not shown).d. In the presence of granisetron, bethanechol (2×10^−6 ^M, added 10 min before start of trace), evoked rhythmic LDC- like pan-colonic propulsive contractions with mid- and distal colon antegrade and bi-directional contractions (rat 2). e. Rhythmic retrograde contractions in distal colon became dominant after 30 min in the presence of granisetron and bethanechol (2×10^−6 ^M, added 20 min before start of trace) (rat 2).

Granisetron (3.8 µM, Figure 6, n = 8) abolished LDCs in 10–20 min. In the presence of granisetron, segmentation activity was prominent in mid and distal colon (Fig. 6b) but in 2 of 8 experiments it was preceded by a 3–5 min period of complete absence of activity (Figure 6c). After addition of bethanechol in the presence of granisetron, rhythmic LDC-like pan-colonic propulsive contractions and concomitant distal retrograde contractions developed (Figure 6d, Table 2). In some experiments, the retrograde contractions became dominant (Figure 6e) and LDC-like activity did not develop.

**Table 2:** Bethanechol-induced motor patterns in the presence of granisetron.

	LDCs before granisetron (n = 8)	In the presence of granisetron and bethanechol	
		LDC-like motor pattern	Retrograde contractions
Propagation length (cm)	10.42±1.89	9.75±1.73	3.10±1.04
Contraction duration (s)	31.33±9.42	96.25±12.69**	33.67±8.14
Relaxation phase duration (s)	12.00±3.46	23.00±3.16*	17.67±7.51
Frequency (cpm)	0.52±0.12	0.44±0.13	0.59±0.24
Velocity (mm/s)	3.7±1.7	1.00±0.20*	1±0.5
Relaxation phase velocity (mm/s)	9.4±3.3	4.30±0.80*	2.1±1.5
Diameter change (cm)	0.42±0.10	0.37±0.04	0.41±0.17

Mean values ± S.D. The length of the colon was 13.8±1.8 cm; the colon diameter was 0.83±0.17 cm (n = 55). * = p<0.05; ** = p<0.001 comparing LDC-like activity after granisetron and bethanechol with spontaneous LDCs before granisetron.

### Segmentation

RPMCs were observed to “break up” into several isolated contractions (∼ 0.5 cm long) such that several (2–5) short-lasting rings of contraction occurred at the same time dividing the colon into segments by contractions that were non-propulsive or propulsive over a very short distance (Figure 5b). Content moved back and forth. Periods of segmentation could occur at any time in the absence and presence of nerve blockade. Segmentation became prominent in the presence of the 5-HT_3_ antagonist granisetron (3.8 µM; Figure 6b).

### Pan-colonic Spatiotemporal Organization of Motor Patterns

The pan-colonic spatiotemporal organization showed rhythmic recurring patterns of motor activity: LDCs followed by ripples or a group of RPMCs followed by LDCs. Importantly, distinct patterns occurred in proximal, mid and distal colon. The LDCs were propulsive throughout the whole colon. The proximal colon, in addition, was dominated by retrograde propagating ripples, and the distal colon by RPMCs propagating in anal direction. 269 periods of 3 min duration were analyzed. Under normal conditions, spontaneous LDCs were associated with RPMCs in the mid and distal colon, 56% of the time (151/269). A pattern of 2–5 RPMCs followed the LDC facilitating clearance of the distal colon (Figure 1d). This pattern would repeat itself in a very similar manner after the subsequent LDCs. A segmentation pattern followed the LDC 30% of the time (75/269) or no other patterns were obvious (43/269, 16%). Ripples almost always followed the LDCs with enhanced diameter changes (Figures 1a,b) and ripples could be seen superimposed on the LDCs in the proximal colon (Figures 4b, 5b).

## Discussion

In recent years, studies on propulsive contractions in the colon of various species have often grouped them together and named them colonic migrating motor complexes (CMMCs) [4], [6], , giant contractions [8], haustral contractions [9], mass peristalsis [9] or “rhythmic propulsive motor complexes” [1]. We report here that distinctly different propulsive motor patterns occurred in the fluid filled rat colon which could not be characterized by a single term. Three dominant colonic motor patterns occurred. Antegrade propagating long distance contractions (LDCs) characterized by a sustained component and a retrograde propagating relaxation phase occurring at a frequency of 0.3–2/min. Antegrade propagating rhythmic propulsive motor complexes [1] (RPMCs) which were ring contractions without a sustained component and propagating with highly variable speeds at 0.3–6/min. In addition, ripples [1], [4], [10] occurred at a constant frequency of 6–10/min; ripples were of low amplitude and often changed direction of propagation, suggesting that they served primarily absorption.

The pan-colonic spatiotemporal organization was as follows: LDCs, interrupted LDCs and tandem contractions started in the very proximal colon and ended in the distal colon. Retrograde propagating ripples were prominent in the proximal colon and almost always followed LDCs. RPMCs almost always occurred simultaneously with LDCs in the distal colon.

LDCs and RPMCs were abolished by TTX, atropine and 5-HT_3_ antagonists, indicating their neurogenic nature. Most recent studies suggest the enteric nervous system to be responsible for the rhythmicity [4]. However in the presence of TTX, very similar motor patterns were evoked by muscarinic receptor stimulation indicating that myogenic control systems exist that guide rhythmic initiation and propagation of propulsive motor patterns.

The present study was performed on fluid-filled colons. Pellets inhabit the mid and distal colon most of the time and the influence of the motor patterns on pellets needs further investigation, as does the influence of pellets on the motor patterns. It was recently noted that pellets make rhythmic propagating motor activity more regular and increase the frequency of the contractions [11].

Are the LDCs similar to CMMCs reported in the literature? CMMCs were almost always studied in flat sheet preparations where contractions are monitored at 3–4 sites. In such preparations it is difficult to distinguish between the various motor patterns identified in the present study where diameter changes are measured all along the colon. Second, the ENS is disrupted, in particular the circumferentially-oriented network of AH neurons [12]. For example, it was reported that the majority of CMMCs start in the mid colon [13] or seen to travel retrograde [11]. Since LDCs propagated antegrade and always start in the proximal colon, it would classify CMMCs as RPMCs. Another example is the effect of blockade of nitric oxide synthesis. It was reported that L-NNA increased the frequency of CMMCs [14] but we report here that L-NNA abolishes LDCs and replaces them with high frequency contractions with features very different from LDCs. It is therefore likely that the flat sheet preparation alters the ENS circuitry sufficiently to markedly affect the features of colonic motor patterns.

### Myogenic Pacemakers

The colonic rhythmic ring contractions with the most constant frequency were the ripples. Ripples persist in the presence of TTX. Their frequency is the same as that of the omnipresent slow wave activity of the rat colon, which is generated by the ICC associated with the submuscular plexus (ICC-SMP) [1], [15]. The ripples often propagate retrograde but are seen to change direction frequently. They are always of low amplitude hence they are not a force in propulsion of colonic content and likely promote absorption, consistent with the interpretation in other studies [6].

Both LDCs and RPMCs occur in a rhythmic manner, the rhythmicity is usually stable within a preparation under certain conditions but it is quite variable when conditions change and variable comparing different preparations. Most often the activity is between 0.3 and 2 cycles/min. Although both motor patterns can be abolished by TTX, this does not prove that the ENS governs the rhythmicity. The fact that in the presence of TTX activities similar to both motor patterns can be evoked by pharmacological means indicates that a myogenic pacemaker is present and may operate under all conditions. The LDC-like contractions that occur in the presence of nerve block indicate that myogenic mechanism of initiation, rhythmicity, propagation, sustained contraction and sudden complete relaxation are present. All these properties of contraction can be orchestrated by slow wave activities [16], [17]. Consistent with this is the occurrence of slow depolarizations during low frequency propagating contraction seen in mouse colon [2], [14]. The laboratories of Jimenez and Takaki have provided strong evidence that ICC associated with the myenteric plexus (ICC-MP) are associated with a low frequency pacemaker that can drive contractions at frequencies from 0.3 to 2 cycles per minute [15], [18]. The ICC-MP may therefore drive all characterized “neurogenic” propulsive activities since LDCs, interrupted LDCs and tandem contractions all occur rhythmically within the same frequency range.

It is important to note that rhythmic contractions are almost always present in the proximal colon, also in the presence of TTX. We speculate that these contractions receive their rhythm from ICC pacemakers, that the contractions excite sensory neurons [19], which initiates a neural program to develop pan-colonic propulsive contractions.

### Neural Control

Serotonin likely plays a critical role in the generation of colonic motor patterns since the 5-HT_3_ antagonist granisetron abolished both fluid-induced and spontaneous LDCs and induced other patterns. Fluid infusion caused a minor distention clearly visible in the spatiotemporal maps, but it would also cause mucosal stroking which likely would release 5-HT from enterochromaffin cells [13] that may activate enteric sensory neurons, which might initiate a neural program to create the LDCs. Since rhythmic propulsive motor activity was shown to persist after removal of the mucosa [13], enterochromaffin cells are unlikely to be essential for the rhythmically occurring LDCs and therefore the generation of the rhythmic LDCs likely involves serotonergic interneurons [20]. Serotonergic interneurons may directly or indirectly affect motor neurons, ICC and smooth muscle cells [21]. In the small intestine, it was shown that 5-HT augments pacemaker activity via 5-HT_3_ receptors on ICC [22]. Serotonergic neurons also have acetylcholine as a neurotransmitter [23]; hence a function of these interneurons is consistent with hexamethonium blocking rhythmic propulsive activities [8]. In our study, not only did granisetron abolish the propulsive LDC, it additionally promoted segmentation motor activity and retrograde contractions. This is consistent with clinical studies finding that granisetron and ondansetron decreased postprandial sigmoid motility, delay in colonic transit, and increased stool consistency in IBS patients with diarrhea [24]. The dramatic effects of the 5-HT_3_ antagonist in the present study would suggest highly successful treatment with 5-HT_3_ antagonists but the success rate for relieving diarrhea is limited [20]. Although the 5-HT_3_-antagonist alosetron reduced diarrhea in some patients and caused constipation in others, most patients did not benefit indicating that mechanisms other than serotonin contributed to motor dysfunction and/or that genetic variation amongst patients in serotonin signaling prevented a uniform action [20], [25], [26].

Cholinergic motor neurons play a critical role in the generation of LDCs and RPMCs since atropine abolished these contractions. Muscarinic acetylcholine receptors are found on smooth muscle cells as well as ICC [27], [28]. Since atropine reduced the frequency of LDCs it is likely that the ICC-MP pacemaker frequency is regulated by muscarinic acetylcholine receptor activation on ICC-MP. Consistent with this is the abundant innervation of ICC-MP by cholinergic nerves [1]. Also consistent is the abnormal colonic motor functions in Hirschsprung’s disease where cholinergic innervation is compromised [29] and the reduced amplitude of mass movement in response to hexamethonium and atropine in the rat *in vivo* [30].

Nitrergic nerves not only provide the relaxation phase preceding the propulsive motor activities in the mid and distal colon, they are an integral part of the neural program that orchestrates the LDCs since L-NNA abolished all of the LDC, not just the relaxation. In the presence of L-NNA, strong rhythmic activity remained at a higher frequency suggesting that nitrergic nerves inhibit pacemaker frequency of ICC-MP. This is consistent with reports that NO donors inhibit ICC-MP activity measured by calcium imaging [31]. Nitrergic innervation is relatively sparse in the proximal colon and increases 260% in the mid colon [32]. This may contribute to the mid colon showing relaxations as observed with interrupted LDCs and tandem contractions. Persistent inhibitory neural activity is still present in the proximal colon since TTX and L-NNA always enhance the amplitude of the ripples.

In the presence of TTX and bethanechol retrograde contractions starting from the distal colon are always present. Without neural blockade, the motor complexes in the distal colon are predominantly antegrade propagating; this suggests that under these conditions a distal pacemaker is inhibited by neural activity, further emphasizing that the dominance of pacemakers in specific regions of the colon is modulated by the ENS.

Enteric neurons other than nitrergic, cholinergic and serotonergic nerves likely play a role in the orchestration of propulsive activities. A prominent inhibitory component in the rat colon is the purinergic innervation [33]. Another candidate for excitatory neurotransmission is substance P [34]. Future studies will reveal more details of the neuronal programs that govern the various motor patterns.

### Sustained Rings of Constriction

The present study shows that a sustained distention/contraction triggers RPMCs. An LDC can proceed distal to the distention/contraction or it can be halted. Hence, the enteric neural programs are intricate neural circuitries of sensory and motor neurons that are sequentially activated to create a complex motor pattern with feedback from content or the contractile state of the colon. This contrasts with a simple reflex where a stimulus evokes ascending contraction and descending inhibition [35]. Sustained rings of constriction occurred in 2/55 preparations; they were present for the entire duration of the experiments. Preliminary experiments showed that such constrictions were common in colons inflamed by TNBS, with as prominent characteristic the initiation of distal RPMCs (Chen and Huizinga, unpublished data). All constrictions were in between mid and distal colon. The constriction has resemblance to Cannon’s ring or Cannon’s sphincter noted in the human colon which is a transient but relatively long lasting constriction occurring at the mid transverse colon, from which contractions can be initiated. Theories as to the origin of sustained constriction at that site are that Cannon's sphincter occurs where there is overlap of the superior and inferior mesenteric nerve plexuses [36]; also, a “neuromuscular imbalance” is proposed [37]. There is no structural evidence of increased density of ICC pacemaker cells in the constricted region in the rat (Wang and Huizinga, unpublished observations).

### Comparison with Human Motor Activity

Is rat colon motor pattern information relevant for the human colon? In contrast to the rat colon, the human colon longitudinal muscle is divided into taeniae and the human colon does not create highly regularly shaped pellets. However, it is not known whether or not this indicates differences in basic motor patterns. There are many similarities. The ICC composition and structure is very similar. Networks of ICC are found in the myenteric and submuscular plexus areas and intramuscular ICC are abundant [38]–[40]. One difference is that the human colon has more septa and hence more ICC-IM associated with septa. At this moment there is no reason to assume that the enteric neuronal circuitry is very different.

The electrical activities recorded from human, dog, mouse and rat colon musculature reflecting properties of ICC pacemaker cells and the musculature have similar features [15], [18], [41]–[44]. All have a slow wave type oscillation, which is most prominent near the submucosal border of the circular muscle and hence likely associated with ICC-SMP. And all have low frequency slow depolarizations near the myenteric plexus with superimposed high intensity spiking and associated forceful contractile activity. In the human, in this area also high frequency oscillations (called membrane potential oscillations (MPOs) by some groups) are recorded but they often occur in bursts and then again show slow transient membrane depolarizations with superimposed oscillations and/or action potentials [42]. The slow depolarizations are most likely originating from ICC-MP [15], [18].

Correlation of the motor patterns described in the present study with *in vivo* motor patterns in humans will require *in vivo* high-resolution manometry in humans which is still in its infancy, but we do know that rhythmic propulsive motor activity is common in humans [45]. Slow transit constipation patients show a paucity of propagating pressure waves and an increase in frequency of retrograde propagating sequences [3]. The present study suggests that a paucity of propagating contractions can indicate a reduction in one of many critical components of enteric neural control, including a reduction in 5-HT_3_ receptor mediated activity. In addition, an abnormal pacemaker network can hinder the initiation of propulsive activities [46], [47], which has been demonstrated in slow transit constipation [48].

In conclusion, high-resolution spatiotemporal mapping of pan-colonic motility reveals distinct motor patterns in proximal, mid and distal colon that may serve simultaneously different functions of the colon such as transit, storage, absorption, feces shaping and excretion. Serotonergic, cholinergic and nitrergic circuits are all essential components of the enteric neural programs that generate propulsive and non-propulsive (promoting absorption) motor activities likely in concert with pacemaker activities from ICC-DMP and ICC-MP.

## Supporting Information

Video S1 **Induced and spontaneous LDCs.** Two LDCs are shown, the first one occurred in response to liquid infusion, the second one occured spontaneously. See boxed area in figure 1a. The colon section in the movie was 7.5 cm long, not visible are the proximal 1.5 cm and the distal 1.5 cm of the colon. The video shows in real time.
(MOV)

Video S2 **The interrupted LDC.** One interrupted LDC is shown. See boxed area in figure 1b. The colon section in the movie was 7.5 cm long, not visible are the proximal 1.5 cm and the distal 1.5 cm of the colon. The video shows in real time.
(MOV)

Video S3 **The tandem contraction.** One tandem contraction is shown. See boxed area in figure 1c. The colon section in the movie was 7.5 cm long, not visible are the proximal 1.5 cm and the distal 1.5 cm of the colon. The video shows in real time.
(MOV)

Video S4 **Rhythmic propulsive motor complexes (RPMCs).** RPMCs are shown followed by a spontaneous LDC. See boxed area in Figure 1d The colon visible in the movie is 9.1 cm long. Not visible are 1.3 cm at the proximal end and 1.4 cm at the distal end that were used for anchoring colon on in and outflow tubes. The video shows in real time.
(MOV)
